# The Prevalence and Severity of Dental Caries Among Pregnant Women in the State of Rio de Janeiro, Brazil

**DOI:** 10.1007/s10995-023-03682-9

**Published:** 2023-06-28

**Authors:** S. Groisman, J. J. de Soet, C. M.C. Volgenant

**Affiliations:** 1 Faculdade de Odontologia da Universidade Federal do Rio de Janeiro, Rio de Janeiro, Brazil; 2 Department of Preventive Dentistry, Academic Center for Dentistry Amsterdam, Vrije Universiteit Amsterdam and University of Amsterdam, Amsterdam, The Netherlands; 3 Department of Cariology, Academic Center for Dentistry Amsterdam, Vrije Universiteit Amsterdam and University of Amsterdam, Amsterdam, The Netherlands

**Keywords:** Dental caries. Oral health. Preventive dentistry. Pregnant women. Clinical study

## Abstract

**Aim:**

To assess in a pilot study the prevalence and severity of dental caries among pregnant women compared to non-pregnant women in the state of Rio de Janeiro, Brazil.

**Materials and methods:**

A cross-sectional, observational study was conducted. Data collection consisted of clinical examinations and general questionnaires concerning oral hygiene habits and recent dental visits of pregnant and non-pregnant women. Prevalence and severity of caries was determined by the CAST index and the CAST severity score. Permission for this study was granted by the National Research Ethics Commission of Brazil. Written informed consent was obtained from all participants.

**Results:**

In total, 67 pregnant women were included (mean age (SD) 25.5 ± 5.4 years) and 79 non-pregnant women (26.0 ± 5.3 years). Mean number of teeth with untreated caries (CAST 4–7) among pregnant women was significantly lower (1.2 ± 1.8) compared to non-pregnant women (2.7 ± 4.0; Mann-Whitney test, p = 0.027). In both groups, 40–60% were in need of curative treatment. There was no significant difference between the two groups in frequency of dental visits (p > 0.05), but pregnant women brushed their teeth more often (Mann-Whitney test, p < 0.001).

**Conclusion:**

Pregnant women in the state of Rio de Janeiro have fewer untreated caries and less severe dental caries compared to non-pregnant women. Still, half of all women in this study are in need of curative treatment for at least one tooth. Effective preventive programs should therefore be developed to stimulate preventive oral care among all women.

**Supplementary Information:**

The online version contains supplementary material available at 10.1007/s10995-023-03682-9.

## Significance

Knowledge of the oral health of pregnant women, with special emphasis on dental caries, in areas with a low socio-economical status (SES) is lacking. This study reports a need for curative dental treatment, which was not significantly higher for non-pregnant women living in the same area with a similar SES. The number of active carious lesions was lower for pregnant women. It is suggested to extend maternal dental care in low-SES communities also for non-pregnant women. Taking care of women’s oral health brings improvement and will also affect their children positively.

## Introduction

The average prevalence of dental caries in Brazil is high. There is great economic disparity in the country (Peres et al., 2011): many residents have a low socio-economic status (SES), which is associated with a higher risk of dental caries (Costa et al., 2012). To improve health care access for people with a low SES, in 1988 Brazil introduced a national health system, Sistema Único de Saúde (SUS), which provides free basic health care. The SUS system includes free basic oral health care to provide people with a low SES the possibility to have regular dental visits. The latter is proven to be associated with improved oral health (Thomson et al., 2010).

Depending on SES, the DMFT index (Decayed, Missed and Filled Teeth) among 24-year-olds in the south of Brazil ranges between 5 and 6; young adults with a high SES have a mean DMFT below 4. The oral health of parents is associated with the prevalence of caries in the primary dentition of their children (Borges et al., 2012; Reisine et al., 2008). Early childhood caries is more prevalent among children of mothers with caries (Paglia et al., 2016). The highest risk for dental caries in Brazil, however, is among women with low SES (Boing et al., 2014). In a national health survey among young Brazilian residents the mean DMFT was 4.25 for 15-19-year-olds and 16.8 for 20-34-year-olds (Ministério-da-Saúde, 2012). In these studies it was noted that the national survey focuses on the richer parts of the country, and reliable data on residents with low SES are not available (Boing et al., 2014). Overall, Boing et al. noted that many studies on the association between oral diseases and demographic characteristics among the Brazilian population are flawed due to design bias. It is therefore not reliable to make general statements about the association between demographic factors and oral health in the Brazilian population.

Dental caries is the primary cause for oral pain during pregnancy (Krüger et al., 2017). Hormones cause the oral environment to change during pregnancy, which may lead to a decrease in salivary pH and buffer capacity at the end of the pregnancy (Laine & Pienihakkinen, 2000). To reduce the risks of oral disease and dental caries for mother and child, it is recommended that women get preventive and curative care during pregnancy. Unfortunately, access to oral health care for pregnant women is reported to be limited in parts of Brazil (Krüger et al., 2017), and 56–67% of the population with a low SES in Brazil does not see a dentist regularly (Araujo et al., 2017; Boccolini & de Souza Junior, 2016; Mullachery et al., 2016). SUS recognizes that all pregnant woman should see a dentist at least once during pregnancy, and the number of dental visits is increasing (Mullachery et al., 2016). This pilot study aimed to compare caries prevalence and the need for dental care among pregnant women participating in already installed care for pregnant women with non-pregnant woman, both visiting healthcare centres in the state of Rio de Janeiro, Brazil.

## Materials and methods

### Study Population

This cross-sectional case-control study was conducted in the state of Rio de Janeiro, Brazil, at public health centres in low-income neighbourhoods of the cities of São Gonsalo, Arraial do Cabo and Resende. Free health care and oral health care was offered to the residents, provided by the SUS system. We randomly included all women aged 18–40 visiting public health centres in the 5-week study period in 2018. Women who reported being pregnant (4 to 7 months) were allocated to the pregnant group, non-pregnant women (self-reported) were allocated to the control group. Exclusion criteria were absence of a written informed consent form and the presence of fixed orthodontics or dentures.

All women in this study came from a low SES location. In a parallel study with a comparable but younger group, the FAS score (family affluence scale) was measured in 169 adolescents. This FAS score averaged 6.40 (SD = 1.46), which is much lower than many other areas in Brazil and Europe (Hobza et al., 2017). Moreover, the per capita income of the cities studied turned out to be significantly lower than elsewhere in Brazil (IBGE, 2018; The-World-Bank, 2020).

### Data Collection

Permission for this study was granted by the National Research Ethics Commission of Brazil (registration number: FR – 156,086). Candidates received verbal and written information about the study and gave written informed consent. Participants were subsequently asked to complete a questionnaire, and in case a participant was illiterate a translator read the questionnaire out loud. The questionnaire contained questions about their oral hygiene and dental visits.

### Clinical Examination

Dental caries was recorded according to the Caries Assessment Spectrum and Treatment (CAST) (de Souza et al., 2012; Frencken et al., 2011). The examinations were performed by three final-year master’s students. Training and calibration of the examiners was conducted at the Federal University of Rio de Janeiro (UFRJ), and training continued until Cohen’s kappa reached values higher than 0.8. The teeth were scored individually by two examiners; next, scores were compared and differences were discussed until consensus was reached. The clinical study was conducted in 2018.

Subjects were examined in an upright position, with a mirror and a headlight to illuminate the mouth. Excess saliva was removed by asking the subject to swallow and the teeth were dried with a cotton roll. The highest CAST score was recorded per tooth according to the CAST index. With the highest CAST score per tooth, the CAST severity score F was calculated per participant (Ribeiro et al., 2018): the more severe the dental caries, the more it weighs in the formula. Depending on the severity score, participants can be classified as mildly (< 1.25), moderately (1.25–6.75) and severely (> 6.75) diseased (Ribeiro et al., 2018).

The need for curative treatment was defined as CAST 4–7, from dentine caries without evident cavitation to abscess or fistula. Per participant it was verified whether they had untreated caries (yes/no) and the number of teeth with untreated caries. In this way, it was possible to assess the number of women that needed curative treatment.

### Statistics

Descriptive statistics were performed by calculating the mean, standard deviation and percentages. A two-sample t-test was used to assess possible differences in age between pregnant and non-pregnant women. Further data had non-normal distribution (Shapiro Wilk test for normality, P < 0.0001), so Mann-Whitney U-tests and chi-square tests were used to assess possible associations. If there was a statistical difference, a post-hoc chi-square test was performed. To assess predictability of the severity score by the other variables, a negative binomial regression analysis was used.

Data analyses were performed using IBM SPSS Statistics for Windows, version 24.0 (IBM Corp., Armonk, N.Y., USA) and the regression analysis was performed using StataCorp (2015). Stata Statistical Software: Release 14. College Station, TX: StataCorp LP. The statistical significance was set at a confidence level of 95%, alpha < 0.05.

## Results

### Description of the Study Population

In total, 67 pregnant women (25.5 ± 5.4 years) and 79 non-pregnant women (26.0 ± 5.3 years) agreed to participate in the study (no statistically significant difference, p = 0.53, t-test). Of all women, 31.7% indicated that they saw a dentist at least twice a year, 22.8% less than twice a year, 15.9% only when experiencing pain, and 29.7% never went to the dentist. There was no significant difference between the two groups in frequency of dental visits (p = 0.27, Mann-Whitney U test) or in dental visits in the last year prior to data collection (p = 0.29, Mann-Whitney U test).

### Need for Treatment

Women who visited a dentist in the previous year had significantly less need for treatment (p = 0.012; Chi-Square test) and fewer teeth with untreated caries (p = 0.009; Mann-Whitney U test); 41.5% of pregnant and 58.5% of non-pregnant women had one or more teeth with untreated caries (no significant difference, p = 0.14; Chi-Square test). Pregnant women had on average fewer teeth in need of treatment (1.2 ± 1.8) than non-pregnant women (2.7 ± 4.0; p = 0.027, Mann-Whitney U test). The need for treatment and the number of teeth with untreated caries did not differ significantly between locations (p = 0.25 and p = 0.054, respectively, both Kruskal-Wallis test).

### CAST Severity Score

The mean CAST severity score on tooth level for pregnant women was significantly lower (8.8 ± 12.5) than for non-pregnant women (20.8 ± 24.1; p = 0.009; Mann-Whitney U test). To assess the influence of the variables age, city of residence, frequency of brushing, recent dental visit and frequency of dental visits on the CAST severity score, a negative binomial regression was performed. A significant influence of pregnancy (yes/no) on the severity score was found (reference group 0 = pregnant women; raw incidence rate ratio (IRR) 2.36, p < 0.01, 95% CI 1.53–3.65; corrected for age and city IRR 2.29, p < 0.01, 95% CI 1.25–3.69; and when corrected for frequency of dental visits and whether a dentist was visited in the last year IRR 2.15, p < 0.01, 95% CI 1.27–3.63).

**Fig. 1:**
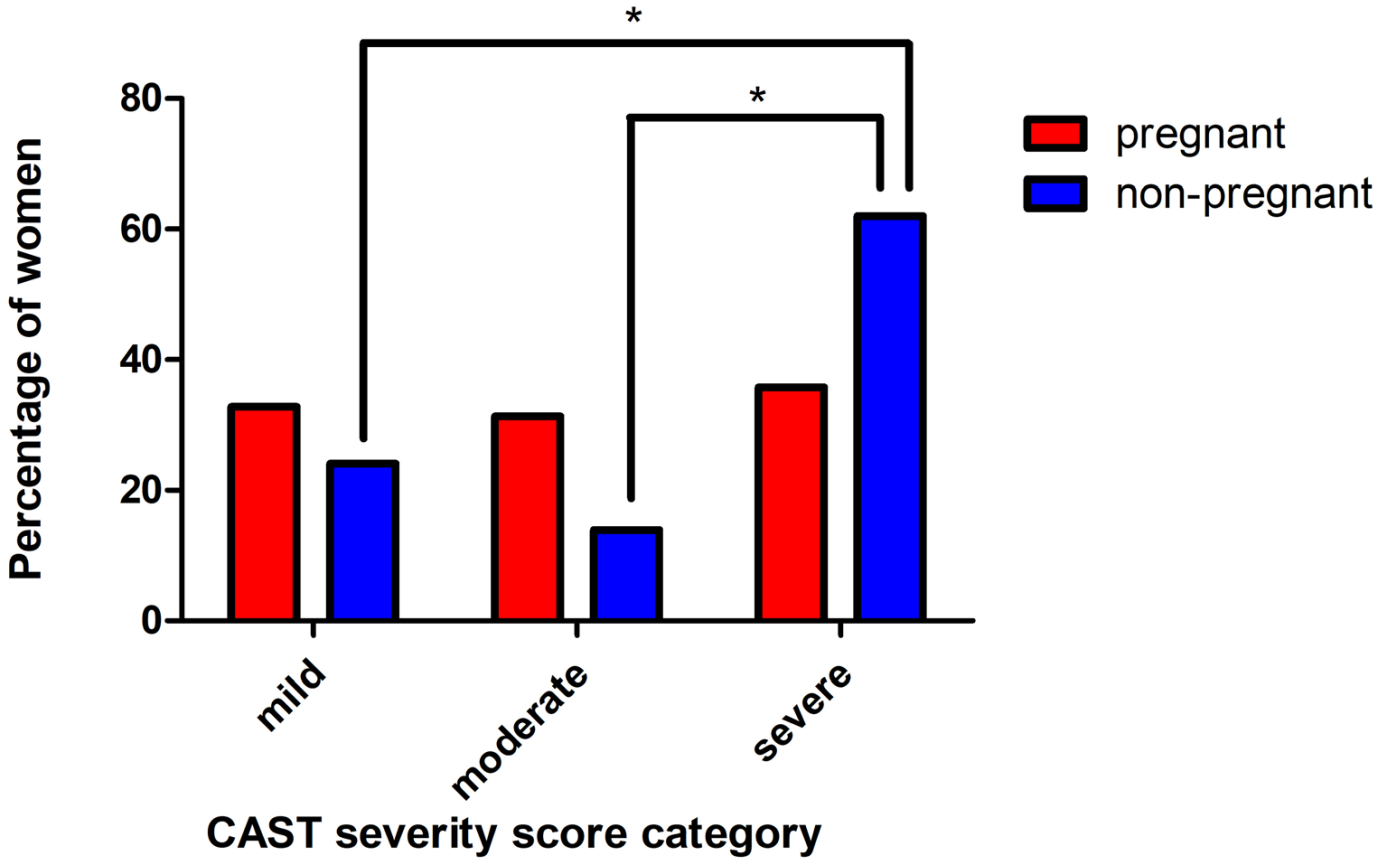
Percentage of women classified into categories by severity score: mild disease (CAST 0–1.25), moderate disease (CAST > 1.25–6.75) and severe disease (CAST > 6.75). * post-hoc chi-square test p = < 0.01)

When classifying each participant in CAST severity categories, more than two-thirds of the women were categorized as having moderate to severe caries. Non-pregnant women were significantly more often in the severe caries group than pregnant women (Fig. 1; chi-square test p < 0.001; post-hoc chi-square test mild vs. severe p = 0.003, moderate vs. severe p < 0.001).

**Fig. 2:**
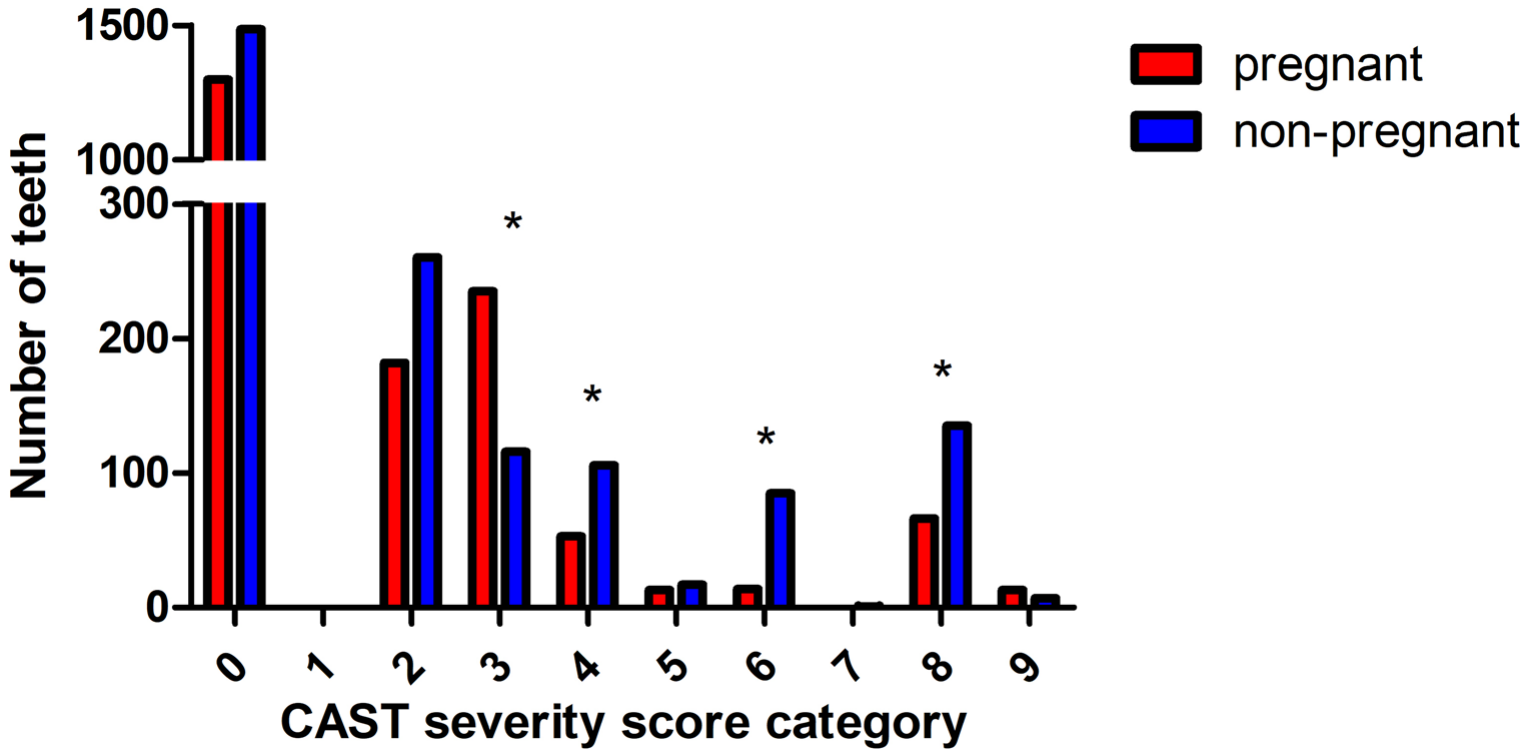
Prevalence of CAST scores in teeth of pregnant and non-pregnant women * Statistically significant between pregnant and non-pregnant women with post-hoc chi-square test, Bonferroni correction alpha = 0.01 (CAST 0 sound; CAST 1 sealant; CAST 2 restoration; CAST 3 enamel changes, without cavitation; CAST 4 caries into dentin; CAST 5 cavitation into dentin, pulp chamber intact; CAST 6 caries with the pulp chamber involved; CAST 7 abscess/fistula; CAST 8 tooth loss because of caries; CAST 9 other)

### CAST Score and Prevalence of Dental Caries

The distribution of the CAST score was different for the two groups (p = 0.032; Mann-Whitney U test); pregnant women had more distinct visual changes in the enamel compared to non-pregnant women (CAST 3; p < 0.001; chi-squared test). However, non-pregnant women had significantly more dentine caries (CAST score 4; p = 0.006; chi-squared test), involvement of the pulp chamber due to cavitation (CAST score 6; p < 0.001; chi-squared test), and tooth loss because of caries (CAST score 8; p < 0.001; chi-squared test) compared to pregnant women.

## Discussion

The severity of caries in this sample from female residents of the state of Rio de Janeiro was found to be lower for pregnant women than for non-pregnant women. This study also reports that both groups were in need of curative treatment, in which non-pregnant women had more teeth that needed curative treatment. The two groups did not differ significantly in age, city of residence, dental visits or brushing frequency.

A higher prevalence of dental caries among pregnant women is frequently observed, possibly due to changes in oral environment (Crozier et al., 2017; Kamate et al., 2017) and delay or absence of professional oral health care (George et al., 2013; Kloetzel et al., 2011). The pregnant women in the present study all received prenatal care, which could explain a higher usage of oral health care (Ruiz et al., 2019). Nevertheless, the results show no significant difference in reported frequency of recent dental visits between pregnant and non-pregnant women, which may be due to women giving ‘desirable’ answers.

The mean number of untreated caries for non-pregnant women (2.0) is similar to previous studies. According to the World Health Organization (WHO), the mean DMFT in 2010 in Brazil was 4.3 (ages 15–19) and 16.8 (ages 35–44) (Malmo-University). The mean number of 0.5 untreated carious teeth among pregnant women is much lower compared to other studies with pregnant women conducted in Brazil, where the mean number of decayed teeth was 2.23–4.25 (Bressane et al., 2011; Krüger et al., 2017; Trindade et al., 2018). There was no significant difference between pregnant and non-pregnant women in need for dental treatment (50% and 60%, respectively). This is within the range of previous studies where the percentage of pregnant women in Brazil with untreated caries varies from 29 to 70% (Krüger et al., 2017; Trindade et al., 2018; Weintraub et al., 2010). These results suggest that frequent visits to a centre for prenatal care stimulates women to improve their oral care.

The current study was conducted in collaboration with health care centres. Consequently, all pregnant women in our study received prenatal care, which is not representative for the entire population of Brazil. Viellas et al. (Viellas et al., 2014) reported that 86% of Brazilian pregnant women seek prenatal care. Ruiz et al. (Ruiz et al., 2019) reported that women who seek prenatal care are more likely to use public dental services during their pregnancy. However, in our study 54.5% of participants reported visiting a dentist, which is similar to the study of Ruiz et al. (Ruiz et al., 2019) but higher than reported by Krüger et al. (Krüger et al., 2017). In other countries, dental visits during pregnancy vary from 16.7 to 90%, depending greatly on social and cultural variables (Krüger et al., 2017; Rocha et al., 2018). In the current study, 13.7% of pregnant women reported not visiting the dentist for financial reasons. This is unusual, as basic oral health care is provided free of charge by SUS, which suggests that women are not well-informed about their privileges.

Previous studies have proven the importance of regular visits to the dentist and frequent tooth brushing, preferably with fluoride toothpaste, in preventing dental caries (Kumar et al., 2016; Marinho et al., 2003; Twetman et al., 2003). The current results, however, do not support the influence of these variables on the caries prevalence for this specific population. The higher number of enamel lesions than dentin lesions in pregnant women suggests that these women are more aware of seeking health care and performing preventive behaviour compared to the non-pregnant women in this study population. Overall improvement in awareness for the possibilities to get access to oral health care in Brazil is needed (Mendes et al., 2017; Moyses et al., 2013).

This study has some limitations. First, 148 women were recruited to participate. That was the maximum number we could recruit during the study timeline. The results indicate that a larger-scale follow-up study is justified, but our results are still clear in the expectation that such an investigation will lead to similar conclusions. A second limitation was that the answers to some questions could not be checked. It was easy for control group participants to falsely report regular dentist visits, which may explain the lack of significance between the control and test groups in this respect.

## Conclusions

In this pilot study, pregnant women in the Brazilian state Rio de Janeiro have fewer untreated caries and less severe carious lesions than non-pregnant women. Half of all women in this study are in need of dental treatment. An effective preventive programme should be developed to stimulate preventive oral care not only among pregnant women, but among all women.

### Electronic Supplementary Material

Below is the link to the electronic supplementary material.

Supplementary Material 1

## Data Availability

On request, the data can be shared using a surfdrive link.

## Code Availability (software application or custom code)

NA.
